# Genome-Wide Association Study of Vertical Jump Performance Among Elite Badminton Players

**DOI:** 10.3390/ijms27062533

**Published:** 2026-03-10

**Authors:** Fevzi Coşkun Sökmen, Anıl Kasakolu, Celal Bulgay, Naoki Kikuchi, Hasan Hüseyin Kazan, Seyrani Koncagul, Yeliz Ay Yildiz, Attila Szabo, Nicola Luigi Bragazzi, Mehmet Ali Ergün

**Affiliations:** 1 Department of General Internal Medicine, Gulhane Faculty of Medicine, University of Health Sciences, Ankara 06010, Türkiye; 2 Graduate School of Natural and Applied Sciences, Ankara University, Ankara 06135, Türkiye; akasakolu@ankara.edu.tr; 3 Faculty of Sports Science, Bingöl University, Bingöl 12000, Türkiye; cbulgay@bingol.edu.tr; 4 Graduate School of Health and Sport Science, Nippon Sport Science University, Tokyo 158-8508, Japan; n.kikuchi@nittai.ac.jp; 5 Department of Medical Biology, Gulhane Faculty of Medicine, University of Health Sciences, Ankara 06010, Türkiye; hasanhuseyin.kazan@sbu.edu.tr; 6 Department of Animal Science, Faculty of Agriculture, Ankara University, Ankara 06135, Türkiye; koncagul@ankara.edu.tr; 7 Faculty of Sports Science, Alanya Alaaddin Keykubat University, Antalya 07425, Türkiye; yeliz.ay@alanya.edu.tr; 8 Faculty of Health and Sport Sciences, Széchenyi István University, H-9026 Győr, Hungary; szabo.attila@sze.hu; 9 Laboratory for Industrial and Applied Mathematics (LIAM), Department of Mathematics and Statistics, York University, Toronto, ON M3J1P3, Canada; 10 Human Nutrition Unit (HNU), Department of Food and Drugs, Medical School, University of Parma, 43125 Parma, Italy; 11 Department of Medical Genetics, Faculty of Medicine, Gazi University, Ankara 06560, Türkiye; aliergun@gazi.edu.tr

**Keywords:** athletes, badminton, countermovement jump, countermovement utilization ratio, genome-wide association study, squat jump

## Abstract

Vertical jump performance is known to be a moderately heritable trait. However, previous studies on sport genetics have largely relied on candidate-gene approaches, which do not adequately reflect the polygenic nature of explosive performance, particularly among elite badminton players. Therefore, the aim of the present study was to identify genetic variants associated with lower-limb explosive performance, assessed via vertical jump measures, among elite Turkish badminton players using a genome-wide association study (GWAS) approach. The present study included 90 elite male (n = 47) and female (n = 43) badminton players, and 557 non-athletic controls sourced from a public database. Performance-related traits were evaluated through countermovement jump (CMJ), squat jump (SJ), and their differential. Genome-wide genotyping was performed using DNA microarrays, and associations were examined using linear mixed models fixed for sex/gender, body mass index, and sport experience. Although no variants reached genome-wide significance (*p* < 1.00 × 10^−7^), 13 single-nucleotide polymorphisms (SNPs) exceeded the suggestive threshold (*p* < 1.00 × 10^−5^). CMJ-associated variants were rs4905767, rs2911702, rs10246591, and rs9842454; SJ-associated variants were rs55817650, rs62318127, rs115197840, rs78317172, and rs35930589; and CMJ–SJ-associated variants were rs34638064, rs6679342, rs4931233, and rs9442615. The present study provides preliminary evidence that lower-limb explosive performance among elite badminton players is polygenic, involving regulatory and signaling pathways rather than single performance genes.

## 1. Introduction

Explosive power, commonly defined as the ability to generate high force in minimal time and often operationalized through the rate of force development, is a key performance attribute in elite sport and is routinely targeted through training [1,2]. Its expression is shaped by multiple biological determinants spanning endocrine status (e.g., circulating testosterone), muscle morphology and fiber characteristics (fast-twitch fiber proportion and cross-sectional area), muscle mass and maximal strength, anthropometric features (e.g., stature and calcaneus length), neuromuscular factors, and muscle–tendon architectural properties (e.g., pennation angle and fascicle thickness/length), as well as reaction time [3,4,5,6,7,8,9,10]. Collectively, these determinants underscore that explosive power is a complex and multifactorial trait rather than a single-parameter capacity.

Vertical jump performance is widely used as a practical and valid indicator of explosive lower-limb power [11]. In this context, countermovement jump (CMJ) and squat jump (SJ) are among the most frequently used assessment tools. Although both quantify lower-limb explosive capability, they differ in mechanical execution and neuromuscular involvement. CMJ incorporates a rapid eccentric phase immediately followed by concentric propulsion, whereas SJ begins in a static semi-squat position, thereby minimizing stretch–shortening cycle (SSC) involvement. Accordingly, the differential between CMJ and SJ (CMJ–SJ), has gained attention as an index of the additional contribution provided by the countermovement [12,13]. It is therefore used to operationalize countermovement-related enhancement in explosive lower-limb performance [14]. Contemporary biomechanical evidence further indicates that the CMJ–SJ differential is primarily explained by augmented muscle activation and reduced muscle slack, with a comparatively smaller contribution from elastic energy storage and return [12].

Beyond biomechanical and training-related determinants, substantial evidence supports a meaningful genetic contribution to explosive performance [3]. Heritability estimates for power-related traits, including vertical jumping ability, range from approximately 49 to 86%, highlighting the importance of inherited factors in shaping inter-individual differences [15]. Early sport genetics research has largely relied on candidate-gene approaches, reporting associations between selected variants and power-athlete status or jump-related phenotypes, including CMJ height [4,15,16,17,18,19,20,21]. For example, Orysiak et al. [22] observed significant CMJ-height differences between athletes with wildtype and mutated alleles of the rs1815739 polymorphism on the actinin alpha 3 (*ACTN3*) gene, and Aleksandra et al. [23] suggested that the rs699 (p.M235T) polymorphism on angiotensinogen (*AGT*) may relate not only to power/strength phenotypes (e.g., SJ/CMJ) but also to training responsiveness. However, despite their biological plausibility, single-nucleotide polymorphisms (SNPs) and even aggregated genotype scores based on a limited set of candidate variants typically explain only a modest proportion of performance variance, often around ~10% for CMJ- and sprint-related outcomes [24].

This limitation is consistent with the highly polygenic architecture of explosive power, whereby numerous variants contribute small individual effects. In line with this perspective, recent studies employing polygenic models have substantially reported greater explanatory power, accounting for up to 26% of the variance in jumping performance and isokinetic strength [25]. Importantly, a recent GWAS-based framework focusing on CMJ training response demonstrated that predictive models integrating genome-wide genetic information (e.g., polygenic score; PGS) with phenotypic indicators can explain up to 62.6% of the inter-individual variance in training-induced CMJ adaptations [26]. Together, these findings support a shift toward hypothesis-free, genome-wide approaches that better capture the complex genetic architecture underlying lower-limb explosive performance.

From a sport-specific perspective, vertical jumping ability is closely linked to sprint acceleration and is a key component in sports characterized by repeated, rapid, and explosive actions, including basketball, volleyball, soccer, and badminton [27,28,29]. Badminton, in particular, is characterized by frequent accelerations and decelerations, rapid multidirectional changes in direction, intermittent jumping and lunging actions, rotational movements, and high reactive demands, collectively placing substantial sprint–power requirements on the lower limbs [30]. Although previous studies have described physical and biomechanical correlates of explosive performance in badminton players [31,32,33], genetic investigations in this population remain scarce. Limited evidence suggests that the rs1815739 polymorphism on the *ACTN3* gene may confer a performance advantage among world-class badminton athletes [34]. Nevertheless, reliance on SNPs is insufficient to reflect the polygenic and multifactorial nature of lower-limb explosive performance, particularly in a sport-specific context such as badminton.

Despite the performance relevance of explosive actions in badminton, more detailed phenotype-specific genome-wide evidence for distinct vertical jump components in elite players remains scarce. Because different jumping tasks partially capture distinct mechanical characteristics and motor-control strategies [14], we evaluated CMJ, SJ, and their differential as separate phenotypes. Accordingly, the aim of the present study was to apply a hypothesis-free, genome-wide approach to identify genetic variants associated with inter-individual differences in these traits in elite Turkish badminton athletes. To the best of our knowledge, this study provides the first phenotype-specific genome-wide evidence in this population, generating candidate *loci* for replication and functional follow-up.

## 2. Results

Lower-limb explosive performance indicators showed mean values of 32.0 ± 6.89 cm for CMJ, 27.5 ± 6.07 cm for SJ, and 4.46 ± 3.15 for their differential. Genetic association analyses indicated that none of the traits (CMJ, SJ, or their differential) reached a genome-wide significance (*p* < 1.00 × 10^−7^).

**Figure 1:**
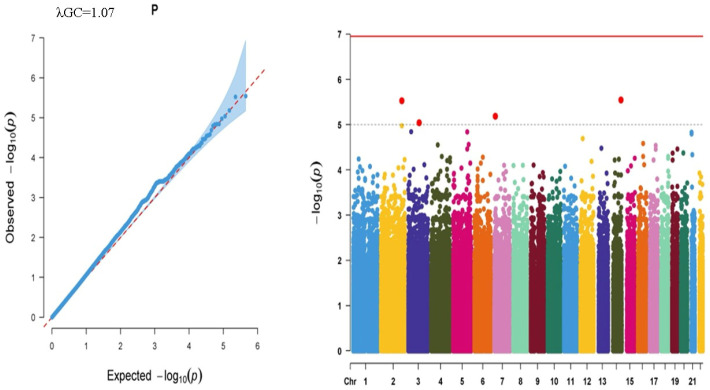
QQ graph showing the observed and expected logarithmic significance and Manhattan Plot illustrating all SNPs on all autosomal chromosomes for the CMJ trait. Dots in the Manhattan Plot represent each SNP on the related chromosomes, colored differentially.

Regarding the suggestive level of significance (1.00 × 10^−5^), four SNPs exceeded the threshold for the CMJ trait (Figure 1). Those SNPs were rs4905767 (NC_000014.9:g.98870526C>T), rs2911702 (intronic variant in long intergenic non-protein coding RNA 1857 (*LINC01857*); NC_000002.12:g.207668344G>T), rs10246591 (intronic variant in PHD finger protein 14 (*PHF14*); NM_001007157.2:c.2772+3393G>A; NC_000007.14:g.11114860G>A), and rs9842454 (intronic variant in guanylate cyclase activator 1C (*GUCA1C*); NM_005459.4:c.204+9326G>A; NC_000003.12:g.108944233C>T). Rs4905767 allele T negatively correlated with CMJ, whereas rs2911702 allele G, rs10246591 allele A, and rs9842454 allele T positively affected CMJ (Table 1). Allele frequencies of those variants were listed as 0.108, 0.014, and 0.221 for rs2911702, rs10246591, and rs9842454, respectively, whereas that of rs4905767 was not documented in the Turkish Genome Project (TGP) database. Collectively, CMJ showed no genome-wide significant associations, but four *loci* reached the suggestive threshold, indicating that CMJ performance is likely influenced by multiple variants with small effects rather than a single major genetic signal. These suggestive findings should be viewed as exploratory and require replication in independent cohorts.

**Figure 2:**
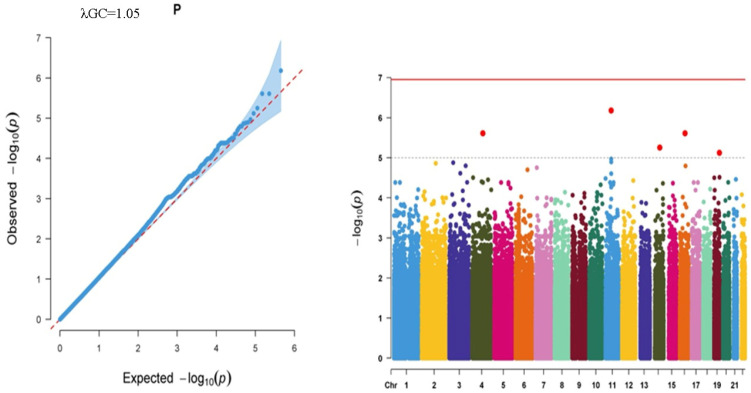
QQ graph showing the observed and expected logarithmic significance and Manhattan Plot illustrating all SNPs on all autosomal chromosomes for the SJ trait. Dots in the Manhattan Plot represent each SNP on the related chromosomes, colored differentially.

Although not reaching genome-wide significance (1.00 × 10^−7^), five SNPs exceeded the suggested threshold (*p* < 1.00 × 10^−5^) for the SJ trait (Figure 2). The significantly associated SNPs were rs55817650 (NC_000011.10:g.56995971T>G), rs62318127 (NC_000004.12:g.106371284G>A), rs115197840 (intronic variant in TOX high mobility group box family member 3 (*TOX3*); NM_001080430.4:c.408+5007A>G; NC_000016.10:g.52458927T>C), rs78317172 (intronic variant in solute carrier family 39 member 9 (*SLC39A9*); NM_018375.5:c.96+1571C>A; NC_000014.9:g.69401036C>A), and rs35930589 (variant in ARPC1A pseudogene 2 (*ARPC1AP2*); NC_000019.10:g.53005024dup). Except the rs35930589 mutated allele (C>CA), rs55817650 allele G, rs62318127 allele A, rs115197840 allele C, and rs78317172 allele A positively correlated with the SJ trait (Table 2). The allele frequencies of those variants were reported to be 0.009, 0.017, 0.022, and 0.174 for rs55817650, rs62318127, rs78317172, and rs35930589, respectively, while that of rs115197840 was not listed in the TGP database. Conjointly, SJ also demonstrated no genome-wide significant associations, but five suggestive *loci* emerged, again pointing to a polygenic architecture with modest effects.

**Figure 3:**
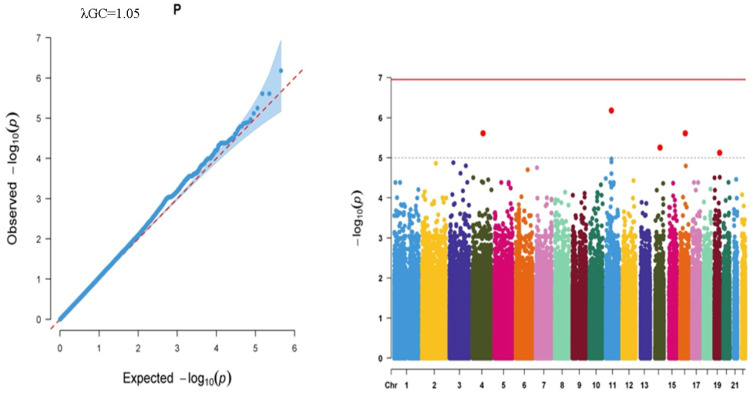
QQ graph showing the observed and expected logarithmic significance and Manhattan Plot illustrating all SNPs on all autosomal chromosomes for the CMJ–SJ trait. Dots in the Manhattan Plot represent each SNP on the related chromosomes, colored differentially.

Finally, for the CMJ–SJ trait, four SNPs were significantly (*p* < 1.00 × 10^−5^) associated with this trait (Figure 3). Those SNPs were rs34638064 (intronic variant in *LOC101928923* gene; NC_000006.12:g.156282855T>C), rs6679342 (NC_000001.11:g.81191250G>A), rs4931233 (intronic variant in *LOC105369715* gene; NC_000012.12:g.30017373G>T), and rs9442615 (intronic variant in protein kinase C zeta (*PRKCZ*); NM_002744.6:c.1575+303T>C; NC_000001.11:g.2175616T>C). Considering the beta values, rs34638064 allele C, rs6679342 allele G, rs4931233 allele G, and rs9442615 allele C positively correlated with the CMJ–SJ trait (Table 3). In the TGP database, the allele frequencies of the variants were determined as 0.144, 0.094, 0.168, and 0.070 for rs34638064, rs6679342, rs4931233, and rs9442615, respectively. In total, the differential between CMJ and SJ yielded four suggestive signals without genome-wide significance, indicating modest genetic contributions to the CMJ–SJ difference.

The predicted effects or linkages of the variants on the mRNA expressions of the related genes (if the variant was in or distally or proximally close to a particular gene) were evaluated using the GTEx Portal (https://www.gtexportal.org/home/; accessed on 16 December 2025, dbGaP Accession phs000424.v10.p2). Accordingly, the results showed that rs9842454, rs55817650, rs78317172, rs35930589, and rs9442615 were associated with altered expression of specific genes in particular tissues (Table 4).

## 3. Discussion

The present study used a GWAS framework to explore associations between common genetic variation and lower-limb explosive performance traits (CMJ, SJ, and their differential) among elite Turkish badminton players. No variant reached the predefined genome-wide significance threshold (*p* = 1.00 × 10^−7^), which is not unexpected given the limited sample size typical of elite-athlete cohorts and the highly polygenic architecture of performance-related traits. Nevertheless, several *loci* showed suggestive associations with CMJ, SJ, and CMJ–SJ-related outcomes, and the allele frequencies of these SNPs were broadly comparable to those reported in the TGP, supporting the plausibility of the observed variation within the general population. In addition, *in silico* predictions suggested that some of these variants may act through regulatory mechanisms in terms of modulation of specific gene expression, consistent with the view that complex athletic phenotypes are often influenced by modest effects distributed across many *loci* rather than by single deterministic performance genes.

Among the CMJ-associated signals, rs9842454 emerged as a notable *locus* due to a pleiotropic regulatory profile. GTEx analyses indicated that this SNP is associated with altered expression of *GUCA1C*, *MORC1*, and the long non-coding RNA *LINC00488* across multiple tissues. However, the functional relevance of this *locus* to CMJ performance remains uncertain, as these genes are primarily characterized in biological contexts that are not directly related to skeletal muscle contraction, motor-unit recruitment, or neuromuscular control. Still, *GUCA1C* has predominantly been studied in retinal phototransduction and visual processing pathways [35]. Therefore, any link between *GUCA1C*-related biology and CMJ performance, potentially via visual perception and visuomotor integration, should be considered indirect and hypothesis-generating, rather than mechanistically established [36]. Similarly, *MORC1* is mainly discussed in the context of stress responsiveness, mood regulation, and epigenetic/transcriptional control in the central nervous system [37,38]. While psychological regulation and stress adaptation could plausibly influence competitive performance, the available evidence does not currently support a direct mechanistic connection to vertical jump capacity. Finally, *LINC00488* remains relatively understudied. Existing reports suggest regulatory roles of this transcript in cell-cycle and gene-expression control [39], but its relevance to explosive neuromuscular output is unknown. Given the limited functional evidence, mechanistic inferences for this *locus* should be considered preliminary and primarily hypothesis-generating.

For SJ performance, the association involving rs55817650 and increased *SSRP1* expression in skeletal muscle appears comparatively more biologically plausible than several other *loci*. SSRP1 has been implicated in myogenin-dependent activation of muscle-specific genes and in processes related to myogenic differentiation and muscle regeneration [40,41]. Conceptually, these pathways align with force-generating capacity and tissue remodeling that may support concentric-dominant tasks such as SJ. In particular, the muscle regeneration/remodeling aspect provides a compelling biological context that could be more directly linked to the concentric force requirements of SJ. However, as with other functional interpretations in this exploratory GWAS, these links remain inferential and should be framed as hypothesis-generating, requiring replication in independent cohorts and further functional validation before any comprehensive conclusions can be drawn. Moreover, variants near *GALNT16* have been reported in a GWAS of musculoskeletal pain phenotypes, suggesting that *O*-linked glycosylation pathways may relate to tissue integrity or mechanical stress tolerance [42]. However, any contribution to jump performance remains inferential. Likewise, genes such as *PLEKHD1*, *ERVV-1*, and *ZNF816* are primarily implicated in immune regulation, transcriptional control, or disease-related processes [43,44,45]. Their association with SJ in the current dataset may reflect broader pleiotropic regulatory effects rather than direct muscle-specific mechanisms and should be interpreted cautiously until replicated and functionally characterized.

For the CMJ-SJ differential-related outcome (operationalized here as CMJ–SJ), reflecting countermovement-related enhancement), the association with rs9442615 highlights a potential role of regulatory elements and intracellular signaling. This SNP is linked to altered expression of *PRKCZ-AS1*, a long non-coding antisense RNA reported to be involved in mitogen-activated protein kinase (MAPK)-related signaling cascades [46]. Because MAPK pathways participate broadly in stress signaling and cellular adaptation, such regulation could plausibly intersect with neuromuscular efficiency; however, the current evidence does not allow a specific SSC mechanism to be inferred. The nearby association with *FAAP20*, a gene involved in cellular resilience and DNA damage repair, is likewise biologically plausible only at a broad level (e.g., general stress tolerance under high training loads) and remains indirect with respect to SSC function [47]. Hence, CMJ–SJ-related interpretations should be regarded as hypothesis-generating pending replication and functional validation.

Notably, none of the identified SNPs have been reported in sport genetic studies with respect to vertical jump or explosive power phenotypes, underscoring both the novelty of the present results and the need for careful interpretation. Rather than implying deterministic performance *loci*, our findings are more consistent with a model in which regulatory, epigenetic, and signaling-related variation contributes subtle effects to neuromuscular coordination and performance expression in an interpretation aligned with contemporary evidence that athletic traits share a complex genetic architecture with broader physiological and neuromotor phenotypes [3]. Taken together, the novelty and non-replication of these *loci* in prior sport-genetics research further highlight the need for responsible communication and ethically grounded interpretation when translating genetic findings into the sporting context [48].

Several limitations should be acknowledged in the present study. First, the relatively small sample size is an inherent challenge in elite-athlete research, likely reducing statistical power to detect genome-wide significant associations and increasing susceptibility to false positives at suggestive thresholds. Nevertheless, regarding the branches of the related athletes of whom the number is limited in a particular population, the problem with small sample size cannot be overcome in sport branch- and population-centric studies. Second, restricting the cohort to Turkish elite badminton players limits generalizability across populations and sport disciplines. Third, explosive performance was evaluated using jump height only; additional kinetic/force–time variables (e.g., peak force, impulse, or rate of force development) could provide a more comprehensive characterization of power-related phenotypes. Finally, the cross-sectional design prevents evaluation of gene-by-training interactions and the extent to which genetic predisposition contributes to long-term adaptation.

## 4. Materials and Methods

### 4.1. Ethics

The present study was approved by the Clinical Research Ethics Committee of Fırat University (Approval no: 2025/15-44). Both written and verbal consent were obtained from all participating athletes or their legal guardians prior to data collection. The present study was carried out in compliance with the ethical principles outlined in the Declaration of Helsinki [49] and followed the Strengthening the Reporting of Genetic Association Studies (STREGA) guidelines, which extend the Strengthening the Reporting of Observational Studies in Epidemiology (STROBE) recommendations [50].

### 4.2. Participants

A total of 90 elite male (n = 47) and female (n = 43) badminton players, affiliated with the Turkish Badminton Federation and actively competing at the international level, participated in the present study. Inclusion criteria required badminton players to hold national team status, to have been ranked among the top three in national competitions, or to have competed in major international events such as the Olympic Games, BWF World Championships, Mediterranean Games, or the European Badminton Championships. The participants had a mean age of 17.11 ± 4.32 years, a mean height of 169.00 ± 9.81 cm, a mean body mass of 61.2 ± 12.0 kg, and an average sports experience of 9.25 ± 3.88 years.

For comparative purposes, genotyping data from 557 healthy individuals obtained from the TGP database (https://tgd.tuseb.gov.tr/; accessed on 13 December 2025) were used as the control group. All participants were of Turkish nationality and of Caucasian origin.

### 4.3. Vertical Jump Tests

Lower-limb explosive performance was assessed using CMJ and SJ. All measurements were obtained with the Optojump System (Microgate, Bolzano, Italy), and jump height was automatically computed and recorded using the manufacturer’s software. Prior to testing, participants completed a standardized warm-up followed by a brief familiarization trial. To minimize upper-body involvement, all jumps were performed with the hands placed on the hips [51].

Participants completed the tests in a fixed order, with the SJ performed first and the CMJ performed second. For each jump test, athletes performed two maximal attempts. A third trial was administered only if either of the first two attempts was technically invalid (e.g., hands were not maintained on the hips) or if the difference between the two attempts exceeded 10%. This discrepancy-based criterion was implemented as a quality-control procedure to ensure within-session consistency, while minimizing unnecessary repetitions and fatigue despite prior familiarization. Attempts were separated by a standardized passive rest interval (≥60 s), and an additional rest period (≥2 min) was provided between the SJ and CMJ tests. The highest valid jump height was used for subsequent analysis [24,52]. CMJ was used to assess explosive power with the contribution of the SSC. Participants started from an upright position with full knee extension, performed a rapid eccentric countermovement, and immediately executed a maximal vertical jump. SJ was used to emphasize concentric explosive force production by minimizing SSC involvement. Participants initiated SJ from a static squat position at approximately 90° knee flexion, maintained this posture for 2–3 s, and then performed a maximal vertical jump without any countermovement. Finally, we calculated the difference between CMJ and SJ height (CMJ−SJ) [12,53].

### 4.4. Genotyping

The present genome-wide investigation was conducted using genomic DNA isolated from peripheral blood samples by a QIAmp Blood Mini Kit (Qiagen, Switzerland) according to the routine protocol of the supplier. Genotyping was performed using an Axiom™ Precision Medicine Diversity Array Kit (Thermo Scientific, Waltham, MA, USA) according to the supplier’s protocol. A total of 752,062 variants *per* participant for 90 players were genotyped, and the quality control (QC) was performed by Plink 1.9 [54] as follows:

i. Minor allele frequency (MAF) was fixed to 0.01 to investigate variants as SNPs;
ii. Genotype call rate (rate of non-missing variants) was selected as 0.90 for further analyses, and imputation was not performed;
iii. Only autosomal chromosomes were evaluated for investigations;
iv. Highly related individual samples were not considered for statistical analyses (>0.45 co-ancestry was eliminated).

At the end of the QC evaluations, a total of 89 players (two of the participants were identical twins, and one of them was eliminated) with 463,849 variants were selected for further investigation.

### 4.5. Statistical Analysis

The statistical analyses were conducted with GEMMA software v.0.98.5 using univariate linear mixed models as follows:

*y* = *Xb* + *Zu* + *wa* + *e*

where y is the phenotype of the relevant trait, X is the model matrix of fixed effects (known environmental effects), including sport experience in years, sex/gender, and body mass index (BMI), b is the effect sizes of the relevant fixed effects, Z is the incidence matrix corresponding to the individual, u is the additive genetic value of each individual (u~*N*(0,Kσ_u_^2^)), K is the relatedness matrix of individuals, w is the marker genotype vector coded with allelic substitution, a is the effect of the relevant variant, and e is the error term of the model (e~*N*(0,Iσ_e_^2^)). In addition to this, population stratification was considered with the relatedness matrix of individuals, and principal components were not evaluated because all individuals represent the same origin.

Additionally, hypothesis tests of the variants were evaluated using Wald’s Significance Test. To reduce the False Discovery Rate (FDR), both Bonferroni Corrected (1.00 × 10^−7^) and suggestive thresholds (1.00 × 10^−5^) were selected [55,56].

Logarithmic *p*-values of observed vs expected significance were given as QQ plots, and the results of genome-wide association analyses were presented as Manhattan Plots for each parameter.

An a priori power analysis was conducted using G*Power 3.1 (Heinrich-Heine-University Düsseldorf, Düsseldorf, Germany) for a linear multiple regression model (random model). The test family was set to “Exact,” and the analysis type was defined as “A priori: Compute required sample size given α, power, and effect size”. A one-tailed test was specified, with an assumed alternative hypothesis effect size of ρ^2^ = 0.20 and a null hypothesis value of ρ^2^ = 0.00. The significance level was set at α = 0.05, and the desired statistical power was 0.97. The model included three predictors.

The analysis indicated that a minimum total sample size of n = 87 participants was required to detect the specified effect size under these parameters. The lower and upper critical R^2^ values were both 0.08, and the achieved (actual) power was 0.97. These results demonstrate that the study design provides sufficient statistical sensitivity to detect a moderate effect size within the specified regression framework. Given the available data records, 90 badminton players were included in the study, and all statistical analyses were conducted on these samples.

## 5. Conclusions

In conclusion, the present findings provide suggestive and hypothesis-generating evidence for a possible genetic contribution to lower-limb explosive performance, highlighting 13 SNPs (rs4905767, rs2911702, rs10246591, rs9842454, rs55817650, rs62318127, rs115197840, rs78317172, rs35930589, rs34638064, rs6679342, rs4931233, and rs9442615) that may be associated with this trait among elite badminton players. GTEx analyses showed that several of these variants were linked to tissue-specific expression of genes like *GUCA1C*, *MORC1*, *LINC00488*, *SSRP1*, *GALNT16*, *PLEKHD1*, *ERVV-1*, *ZNF816*, *PRKCZ-AS1*, and *FAAP20*, particularly in skeletal muscle, neural, and vascular tissues.

Future studies should replicate these findings in larger, multi-center, and multi-ethnic cohorts and evaluate the biological relevance of the identified *loci* through functional validation. Longitudinal training-response designs may help clarify whether these variants influence adaptation trajectories over time, while integrative multi-omics approaches (e.g., transcriptomics, epigenomics, proteomics, metabolomics, and microbiomics) could provide mechanistic insights. In addition, incorporating polygenic models may improve our understanding of how multiple variants collectively contribute to lower-limb explosive performance and may enhance the translational potential of these findings.

## Figures and Tables

**Table 1:** Variants and their details whose significance levels were below the suggested threshold (*p* = 1.00 × 10^−5^) for the CMJ trait.

Variant	Chromosome	Position	Allele	Allele Frequency	Beta Value	Standard Error	*p* Value
rs4905767	14	98870526	T	0.444	−3.758	0.742	2.9 × 10^−6^
rs2911702	2	207668344	G	0.108	6.655	1.317	3.0 × 10^−6^
rs10246591	7	11114860	A	0.025	12.083	2.492	6.6 × 10^−6^
rs9842454	3	108944233	T	0.278	3.900	0.819	9.1 × 10^−6^

**Table 2:** Variants and their details whose significance levels were below the suggested threshold (*p* = 1.00 × 10^−5^) for the SJ trait.

Variant	Chromosome	Position	Allele	Allele Frequency	Beta Value	Standard Error	*p* Value
rs55817650	11	56995971	G	0.151	6.109	1.124	7.0 × 10^−7^
rs62318127	4	106371284	A	0.019	14.224	2.788	2.5 × 10^−6^
rs115197840	16	52458927	C	0.019	14.224	2.788	2.5 × 10^−6^
rs78317172	14	69401036	A	0.025	12.142	2.483	5.6 × 10^−6^
rs35930589	19	53005023	CA	0.130	−5.698	1.184	7.6 × 10^−6^

**Table 3:** Variants and their details whose significance levels were below the suggested threshold (*p* = 1.00 × 10^−5^) for the CMJ–SJ trait.

Variant	Chromosome	Position	Allele	Allele Frequency	Beta Value	Standard Error	*p* Value
rs34638064	6	156282855	C	0.123	2.829	0.525	8.0 × 10^−7^
rs6679342	1	81191250	G	0.056	4.410	0.884	3.8 × 10^−6^
rs4931233	12	30017373	G	0.217	2.687	0.552	6.2 × 10^−6^
rs9442615	1	2175616	C	0.056	3.763	0.794	1.0 × 10^−5^

**Table 4:** Effects or linkage of the SNPs on the expression of specific genes according to the GTEx Portal.

Variant	Affected Gene	Effect/Linkage	Tissue	Normalized Effect Size	*p* Value
rs9842454	*GUCA1C*	Down-regulation	Testis	−0.22	0.000079
	*MORC1* ^a^			−0.55	3.6 × 10^−11^
	*LINC00488*		Brain-Frontal cortex	−0.25	0.000094
rs55817650	*SSRP1* ^b^	Up-regulation	Skeletal muscle	0.44	3.5 × 10^−7^
rs78317172	*GALNT16* ^c^	Up-regulation	Coronary artery	1.4	0.0000063
			Adipose	0.91	2.9 × 10^−8^
	*PLEKHD1* ^d^	Down-regulation		−0.62	1.4 × 10^−8^
rs35930589	*ERVV-1* ^e^	Up-regulation	Thyroid	0.45	1.3 × 10^−10^
	*ZNF816* ^f^			0.17	0.000039
rs9442615	*PRKCZ-AS1* ^g^	Up-regulation	Adipose	0.32	6.9 × 10^−9^
			Artery-Tibial	0.37	4.5 × 10^−8^
	*FAAP20* ^h^			0.53	1.1 × 10^−11^
			Artery-Aorta	0.60	3.8 × 10^−12^
			Brain-Cerebellar hemisphere	0.87	8.9 × 10^−11^
			Skeletal muscle	0.42	1.5 × 10^−8^
			Heart-Left ventricle	0.47	8.6 × 10^−7^

Note: Only selected tissues were listed in the table. ^a^ MORC family CW-type zinc finger 1. ^b^ Structure-specific recognition protein 1. ^c^ Polypeptide *N*-acetylgalactosaminyltransferase 16. ^d^ Pleckstrin homology and coiled-coil domain containing D1. ^e^ Endogenous retrovirus group V member 1, envelope. ^f^ Zinc finger protein 816. ^g^ PRKCZ antisense RNA 1. ^h^ FA core complex associated protein 20.

## Data Availability

Data are available for research purposes upon reasonable request to the corresponding authors.
